# Medication for opioid use disorder among adolescents entering specialty treatment for opioid use disorder and trends in the US, 2017–2022

**DOI:** 10.1016/j.addbeh.2025.108538

**Published:** 2025-10-29

**Authors:** Jesse S. Boggis, Thadryan Sweeney, Lisa A. Marsch, Wesley J. Marrero, Kenneth A. Feder, Erika L. Moen

**Affiliations:** a The Dartmouth Institute for Health Policy and Clinical Practice, Geisel School of Medicine, Dartmouth College, Lebanon, NH; b Center for Technology and Behavioral Health, Geisel School of Medicine, Dartmouth College, Lebanon, NH; c Department of Biomedical Data Science, Geisel School of Medicine, Dartmouth College, Lebanon, NH; d Thayer School of Engineering, Dartmouth College, Hanover, NH; e Department of Mental Health, Johns Hopkins Bloomberg School of Public Health, Baltimore, MD

**Keywords:** Opioid use disorder, Medication for opioid use disorder, Heroin, Opioids, Speciality treatment for substance use, Adolescent medicine

## Abstract

**Background:**

Professional societies recommend that adolescents with opioid use disorder (OUD) receive medication for opioid use disorder (MOUD). This cross-sectional study examined the association between adolescent specialty treatment episodes for OUD with planned MOUD use compared to adults over time.

**Methods:**

We used data on first episodes of specialty treatment for OUD (n = 671,183) from the Treatment Episode Data Set – Admissions, a national database of publicly funded treatment programs in the US Admissions occurred between 1/2017–12/2022. The primary exposure was being adolescent (15–17 years). The main outcome was planned MOUD use, defined as having MOUD in a treatment plan.

**Results:**

Adolescent specialty treatment episodes for OUD were significantly less likely to have planned MOUD use than adults (aOR 0.05, 95% CI, 0.02–0.09). Linear combination tests of the interaction between age group and year confirmed that adolescent episodes were significantly less likely to have planned MOUD use than adults across all years. In 2021 and 2022 this disparity narrowed slightly. In 2021, adolescent episodes had 10% of the adjusted odds of planned MOUD use compared to adults in 2017 (95% CI, 0.07–0.15). In 2022, adolescent episodes had 9% of the adjusted odds of planned MOUD use compared to adults in 2017 (95% CI, 0.06–0.11).

**Conclusion:**

Adolescents entering specialty treatment for OUD had significantly lower odds of planned MOUD use than adults. The relatively smaller difference between adolescents and adults in recent years suggests a potential trend toward greater MOUD access, though future research is needed to understand access barriers.

## 1. Introduction

Adolescents have not been spared in the US overdose crisis. In 2023, 708 adolescents lost their lives from drug overdose, with 79 % of deaths involving opioids and 97 % of opioid involved deaths involving fentanyl (Panchal et al., 2025). The proliferation of fentanyl in the illicit drug supply in pressed counterfeit pills resembling prescription opioids may account for the rise in fatalities as adolescents are more likely to use this pill form, potentially unaware that they are using fentanyl (Palamar et al., 2022; Tanz et al., 2022). Consequently, ensuring access to evidence-based treatment for opioid use disorder (OUD) for adolescents is an urgent public health priority (Committee on Substance Use and Prevention, 2016). Medication for opioid use disorder (MOUD) is the standard of care for treating OUD in adolescents (Committee on Substance Use and Prevention, 2016; Kampman and Jarvis, 2015). However, previous national studies have found adolescents have experienced severely limited access to MOUD (Feder et al., 2017; Welsh et al., 2022).

This study examines first episodes of treatment for OUD conducted at specialty substance use treatment programs across the US from 2017 to 2022. Our objective was to examine the proportion of adolescent treatment episodes for OUD with planned MOUD use compared to adults over time. This study utilized the most recent data available to build upon a 2013 analysis which found only 2.4 % of adolescents entering specialty treatment for heroin had planned MOUD use as compared to 26.3 % of adults and only 0.4 % of adolescents in specialty treatment for prescription opioids had planned MOUD use as compared to 12.0 % of adults (Feder et al., 2017). Given the increased volatility of the illicit drug market in recent years, we sought to understand if the odds of all planned MOUD use in specialty treatment for OUD has changed over time for adolescents and adults.

## 2. Methods

### 2.1. Data source and study cohort

This study used national cross-sectional data from the Treatment Episode Data Set – Admissions (TEDS-A) collected by the Substance Use and Mental Health Services Administration across 2017 to 2022. TEDS is a data repository collected annually by all states receiving public funding for the purpose of monitoring public and private substance use treatment systems. The unit of analysis is treatment admission episodes to either state-licensed or certified substance use treatment facilities. This study was exempt from the human subjects’ ethical research requirements as it involved secondary analysis of deidentified public-use data. We followed the Strengthening the Reporting of Observational Studies in Epidemiology (STROBE) reporting guidelines for cross-sectional studies (von Elm et al., 2007).

Our sample was restricted to adolescent (15 to 17 years) and adult (≥18 years) first episodes of specialty treatment for OUD, defined as episodes where persons were treated primarily for “heroin” or “other opiates and synthetics” to align with the previous 2013 analysis (Feder et al., 2017).

In total, we included data from 38 US states, whereas data from twelve US states, the District of Columbia (DC), and Puerto Rico were not included in the analysis. Delaware, Florida, New Mexico, South Carolina, West Virginia, and Puerto Rico had over half of their MOUD data missing for either adolescents and/or adults across all years and were not included in the analysis. Idaho, Montana, and Oklahoma reported 0 to 1 treatment episodes with MOUD across all years for both adolescents and adults, leading to their exclusion. Connecticut, New Hampshire, and DC reported only one treatment episode for an adolescent across all years and were excluded from the analysis as we assumed this to be a data collection error consistent with prior literature (Solomon et al., 2022). Additionally, data from Oregon and Washington were not reported in all years examined and were excluded from the analysis.

## 3. Measures

The primary outcome variable was planned MOUD use, defined as whether methadone, buprenorphine, and/or naltrexone were a part of a treatment plan at the first specialty treatment episode (yes or no). The primary exposure variable of interest was being of adolescent or adult age at the first specialty treatment episode. We relied on The Model for Analysis of Population Health and Health Disparities as well as previous literature to adjust for multilevel factors potentially related to planned MOUD use (Feder et al., 2017; Warnecke et al., 2008).

### 3.1. Statistical analysis

Prevalence of planned MOUD use for adolescents and adults was estimated for each year. We tested the significance of the effect of adolescence on planned MOUD use during the first specialty treatment episode in every year. An unadjusted comparison of proportions was first used followed by a mixed-effects logistic regression with a random intercept for the treatment program state. The model adjusted for year, referral source, treatment program type, sex, race, ethnicity, housing status, primary opioid use reported at admission, and the interaction between year and age group. A baseline model adjusting for these same covariates excluding the interaction between year and age group was also developed. We then fit a post-hoc linear combination model to assess the interaction between age group and year on planned MOUD use for each year. Effect size was reported using adjusted odds ratio derived from the models and accompanying 95 % confidence intervals. Variables with a 2-sided *p*-value significance threshold of less than 0.05 were considered significant. Variables in the model included 0–7.2 % missing values. They were imputed using Multiple Imputation by Chained Equations (MICE), an approach appropriate in not-at-random cases for low to moderate missing values (van Buuren and Groothuis-Oudshoorn, 2011; Pereira et al., 2024). All analyses were performed using R Version 4.2.3 (R, 2023).

## 4. Results

In total, there were 669,000 first specialty treatment episodes for OUD with adults and 2,183 with adolescents from January 2017 to December 2022 across the included 38 states. Both age groups were mostly male and White. The most common referral source for adults (62.4 %) and adolescents (37.7 %) was by an individual other than a friend or family member, including self-referral. While most clients were entering ambulatory treatment, 72.0 % of adults were entering ambulatory treatment compared with 61.7 % of adolescents. Additionally, 13.3 % of adults were entering rehab/residential treatment compared to 32.7 % of adolescents, and 14.7 % of adults were entering 24-hour free-standing residential detoxification compared to 5.7 % of adults. The second most common referral source for both groups came from the criminal legal system, comprised of 14.1 % of adults and 22.3 % of adolescents. Primary substance use at admission was different between groups, with 66.9 % of adults having reported heroin use at admission compared to 25.8 % of adolescents (Table 1).

In the unadjusted comparison, only 7.3 % of adolescents’ first specialty treatment episodes for OUD planned MOUD use compared to 46.0 % of adult first specialty treatment episodes for OUD across the six years. In the adjusted model with the interaction, first specialty treatment episodes for OUD with adolescents had 5 % of the adjusted odds of planned MOUD use compared to adults (95 % CI, 0.02–0.09) (eTable 1).

**Fig. 1.**
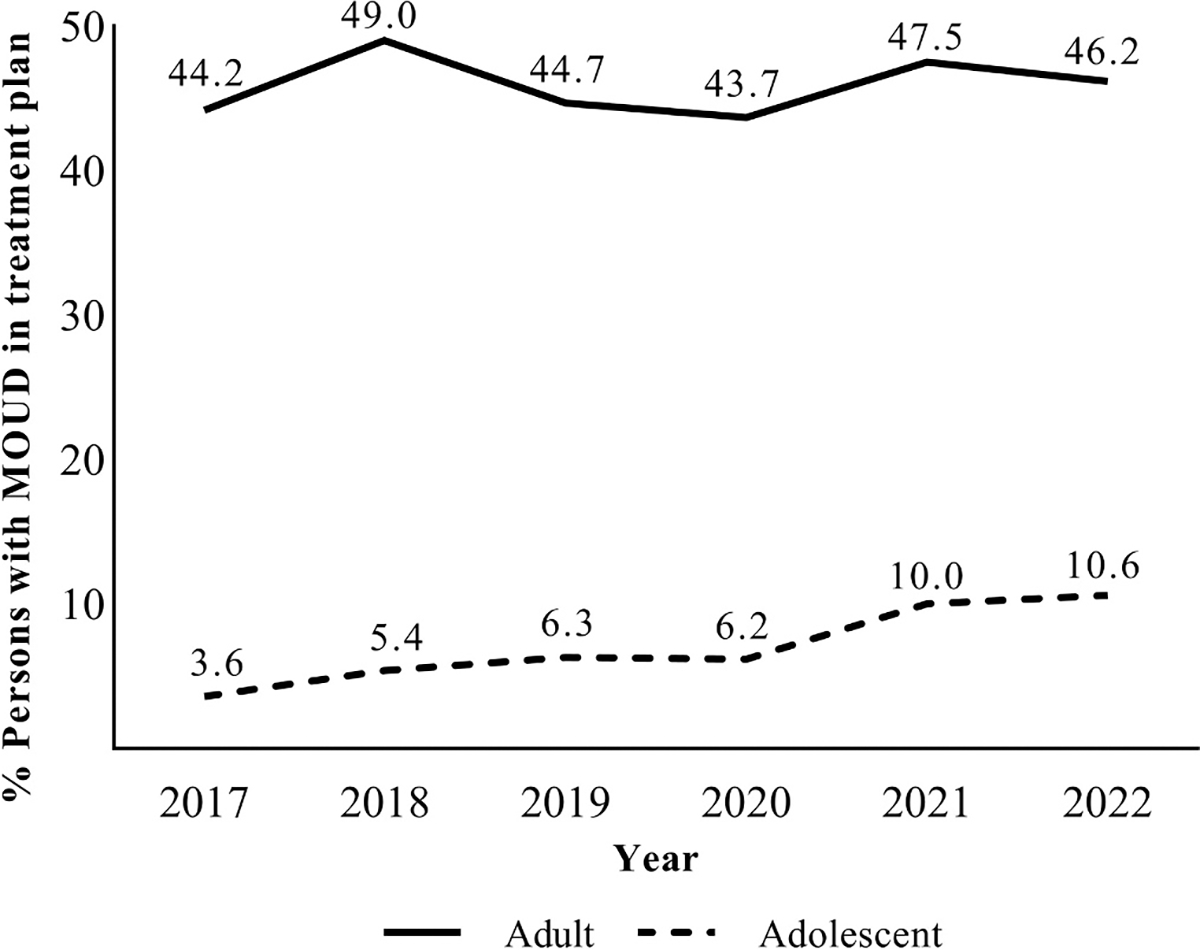
Prevalence of medication for opioid use disorder (MOUD) in a treatment plan among adolescents and adults admitted to US specialty treatment programs for opioid use disorder, 2017–2022.

From 2017 to 2021, we found a persistent gap in MOUD treatment between adults and adolescents (Fig. 1). Post-hoc linear combination tests confirmed that adolescents had significantly lower odds of planned MOUD use during the first specialty treatment episode for OUD from 2018 to 2022, when compared with adults in 2017 (p < 0.001). In 2020, first specialty treatment episodes for OUD with adolescents had 7 % of the odds of planned MOUD use compared to adults in 2017 (95 % CI, 0.04–0.11). In 2021 and 2022 this disparity narrowed slightly. In 2021, adolescent episodes had 10 % of the adjusted odds of planned MOUD use compared to adults in 2017 (95 % CI, 0.07–0.15). In 2022, adolescent episodes had 9 % of the adjusted odds of planned MOUD use compared to adults in 2017 (95 % CI, 0.06–0.11) (eTable 2).

## 5. Discussion

In this cross-sectional study, we found that adolescents had 5 % of the adjusted odds of having planned MOUD use compared to adults entering specialty treatment for OUD. However, our findings indicate modest improvements in adolescent MOUD access since the 2013 analysis conducted (Feder et al., 2017).

We found a pervasive treatment gap in having planned use of evidence-based medicine for OUD by age group despite the heightened prevalence of overdose deaths among adolescents. Buprenorphine is the only MOUD approved by the US Food and Drug Administration for people 16 years and older, while methadone and naltrexone are approved for patients 18 years and older. Health care provider comfort and willingness to prescribe MOUD off-label for adolescents younger than 16 or 18 years old may limit treatment access for adolescents with OUD (Committee on Substance Use and Prevention, 2016; Kampman and Jarvis, 2015). Prior literature has found many additional barriers to adolescent MOUD access including insurance reimbursement, legal consultation, workflow constraints, and lack of education or training among providers, which may help to explain this disparity (McCarty et al., 2022; Knudsen et al., 2024). Given the low rates of adolescent planned MOUD use in 2013, the increase to 7.3 % by 2017–2022 represents an improvement relative to adults, potentially reflecting a meaningful shift in treatment practices for adolescents with OUD. Addressing barriers to access will be essential to make significant improvements in adolescent MOUD use for the future.

### 5.1. Limitations

The TEDS data set does not collect MOUD initiation in office-based treatment. Therefore, our results reflect the MOUD treatment gap between adults and adolescents at the first specialty treatment episode only. The TEDS variable for age at admission is categorical and not continuous. Thus, our inferences are limited to one category of adolescents aged 15 to 17 years, and other age groupings to define adolescents (ex. 15 to 19 years) could not be studied. TEDS does not measure the severity of OUD which may help clinicians determine whether to prescribe MOUD. TEDS combines all MOUD into one variable, thus we are not able to ascertain differences between medications. Twelve US states, DC, and Puerto Rico were excluded, limiting our inferences to our sample. Additionally, while we relied on MICE to impute missing data, this approach may not be appropriate for other data sets. Finally, TEDS captures whether MOUD was a part of the client’s treatment plan only and does not capture whether the client used MOUD, further confining implications of our results to other studies examining planned MOUD use.

## 6. Conclusions

This cross-sectional study found that first specialty treatment episodes for adolescents with OUD had a significantly lower likelihood of planned MOUD use compared to first specialty treatment episodes for adults with OUD. While this treatment gap has remained significant over time, in 2021 and 2022, the gap has narrowed slightly. Our findings draw attention to the importance of investing in clinical practice and policy interventions to expand adolescent access to evidence-based medicine and save lives.

## Supplementary Material

1

## Figures and Tables

**Table 1:** Selected characteristics of first specialty treatment episodes for heroin or other opiates and synthetics among adults and adolescents in the United States, 2017–2022.

	Adult (N = 669,000)	Adolescent (N = 2,183)

Characteristic	N (%)	N (%)

**Medication for Opioid Use Disorder**		
No	336,625 (50.3 %)	1,975 (90.5 %)
Yes	307,570 (46.0 %)	159 (7.3 %)
**Sex**		
Female	260,868 (39.0 %)	906 (41.5 %)
Male	407,834 (61.0 %)	1,274 (58.4 %)
**Race**		
White	462,796 (69.2 %)	1,458 (66.8 %)
Black or African American	98,218 (14.7 %)	158 (7.2 %)
Asian, Pacific Islander, or Native Hawaiian	6,457 (1.0 %)	28 (1.3 %)
Two or more races	15,330 (2.3 %)	110 (5.0 %)
Other single race	52,960 (7.9 %)	296 (13.6 %)
American Indian or Alaskan Native	9,109 (1.4 %)	57 (2.6 %)
**Ethnicity**		
Hispanic or Latino	95,564 (14.3 %)	645 (29.5 %)
**Treatment service**		
Ambulatory (detoxification, intensive and non-intensive outpatient)	481,712 (72.0 %)	1,346 (61.7 %)
Detox, 24-hour (free-standing residential and hospital inpatient)	98,562 (14.7 %)	124 (5.7 %)
Rehab/residential (hospital non-detox, long term, and short term)	88,726 (13.3 %)	713 (32.7 %)
**Referral source**		
Alcohol/drug use care provider	42,586 (6.4 %)	122 (5.6 %)
Criminal legal system	94,197 (14.1 %)	487 (22.3 %)
Employer/Employee Assistance Program	1,208 (0.2 %)	1 (0.0 %)
Individual (includes self-referral)	417,589 (62.4 %)	822 (37.7 %)
Other community referral	64,129 (9.6 %)	368 (16.9 %)
Other health care provider	37,535 (5.6 %)	261 (12.0 %)
School	318 (0.0 %)	94 (4.3 %)
**Living arrangement**		
Dependent living	105,591 (15.8 %)	1,370 (62.8 %)
Independent living	430,827 (64.4 %)	728 (33.3 %)
Unhoused	86,843 (13.0 %)	26 (1.2 %)
**Primary opioid use reported at admission**		
Heroin	447,722 (66.9 %)	563 (25.8 %)
Other opiates and synthetics	221,278 (33.1 %)	1,620 (74.2 %)

Note: Data represent characteristics at the first treatment episode and not individual patients.

Adolescents = Aged 15–17 years; Adults = Aged 18 years or older. Long term = more than 30 days; short term = less than 30 days. Criminal legal system = Court/criminal justice referral/Driving Under the Influence (DUI) and Driving While Intoxicated (DWI) referrals.

## Data Availability

Data used for this study are available here: https://www.samhsa.gov/data/data-we-collect/teds-treatment-episode-data-set/datafiles. The code for this manuscript will be made available upon request.

## Appendix A. Supplementary data

Supplementary data to this article can be found online at https://doi.org/10.1016/j.addbeh.2025.108538.
