# Supplementary material for: Medication for opioid use disorder among adolescents entering specialty treatment for opioid use disorder and trends in the US, 2017–2022
Source: Addict Behav. Author manuscript; Available in PMC 2026 Aug 5. (PMC13441448; doi:10.1016/j.addbeh.2025.108538)
Supplement: Supplementary file 1 [file NIHMS2185853-supplement-1.docx]

**eTable 1**

Adjusted Odds Ratios (aORs) of having planned medication for opioid use disorder (MOUD) as an adolescent admitted to US specialty treatment programs for treatment of opioid use disorder and other characteristics.

| **Characteristic** | **Baseline**  aOR (95% CI) | **Interaction**  aOR (95% CI) |
| --- | --- | --- |
|  |  |  |
| **Primary Exposure** |  |  |
| Adolescent vs Adult | 0.08 (0.06–0.10) | 0.05 (0.02–0.09) |
| **Year** |  |  |
| 2018 vs 2017 | 1.07 (1.04–1.10) | 1.07 (1.04–1.10) |
| 2019 vs 2017 | 0.90 (0.88–0.93) | 0.90 (0.87–0.93) |
| 2020 vs 2017 | 1.00 (0.97–1.04) | 1.00 (0.97–1.04) |
| 2021 vs 2017 | 1.40 (1.35–1.46) | 1.40 (1.35–1.46) |
| 2022 vs 2017 | 1.63 (1.57–1.70) | 1.63 (1.56–1.70) |
| **Sex** |  |  |
| Female vs Male | 1.03 (1.01–1.06) | 1.03 (1.01–1.06) |
| **Treatment Setting** |  |  |
| Rehab/residential vs Detox, 24-hour | 0.51 (0.49–0.54) | 0.51 (0.49–0.54) |
| Ambulatory vs Detox, 24-hour | 6.72 (6.51–6.93) | 6.72 (6.51–6.93) |
| **Race** |  |  |
| Black or African American vs White | 1.00 (0.97–1.03) | 1.00 (0.97–1.03) |
| Asian, Pacific Islander, or Native Hawaiian vs White | 1.03 (0.93–1.13) | 1.03 (0.93–1.14) |
| Two or more races vs White | 1.07 (1.00–1.14) | 1.07 (1.00–1.14) |
| Other singe race vs White | 1.35 (1.29–1.42) | 1.35 (1.29–1.42) |
| American Indian or Alaskan Native vs White | 1.15 (1.06–1.25) | 1.15 (1.06–1.25) |
| **Ethnicity** |  |  |
| Hispanic or Latino vs Non-Hispanic or Latino | 0.94 (0.91–0.98) | 0.94 (0.91–0.98) |
| **Referral Source** |  |  |
| Criminal legal system vs Alcohol/drug use care  provider | 0.15 (0.15–0.16) | 0.15 (0.15–0.16) |
| Employer/EAP vs Alcohol/drug use care provider | 0.29 (0.23–0.37) | 0.29 (0.23–0.37) |
| Individual (includes self-referral) vs Alcohol/drug  use care provider | 2.07 (1.99–2.16) | 2.07 (1.99–2.16) |
| Other community referral vs Alcohol/drug use care  provider | 0.63 (0.60–0.66) | 0.63 (0.60–0.66) |
| Other health care provider vs Alcohol/drug use care  provider | 0.98 (0.92–1.04) | 0.98 (0.92–1.04) |
| School vs Alcohol/drug use care provider | 0.55 (0.37–0.80) | 0.55 (0.37–0.82) |
| **Housing Status** |  |  |
| Unhoused vs Dependent living | 0.73 (0.71–0.76) | 0.73 (0.71–0.76) |
| Independent living vs Dependent living | 1.35 (1.32–1.39) | 1.35 (1.32–1.39) |
| **Primary opioid use reported at admission** |  |  |
| Other opiates and synthetics vs Heroin | 0.83 (0.81–0.85) | 0.83 (0.81–0.85) |
| **Interaction: Year × Adolescent Age Group** |  |  |
| 2018 × Adolescent | – | 1.54 (0.62–3.84) |
| 2019 × Adolescent | – | 2.06 (0.82–5.18) |
| 2020 × Adolescent | – | 1.48 (0.53–4.11) |
| 2021 × Adolescent | – | 2.20 (0.86–5.64) |
| 2022 × Adolescent | – | 1.86 (0.80–4.37) |

Abbreviations: EAP, employee assistance program.

Note: Criminal Legal System includes Court/criminal justice referral/ Driving Under the Influence (DUI) and Driving While Intoxicated (DWI) referrals.

**eTable 2**

Adjusted Odds Ratios (aORs) of planned medication for opioid use disorder (MOUD) among adolescents compared with adults admitted to specialty treatment programs for treatment of opioid use disorder in 2017, by Year.

| **Characteristic** | **aOR (95% CI)** | **P-value** |
| --- | --- | --- |
| **Year of admission** |  |  |
| 2018 | 0.07 (0.04–0.11) | <0.001 |
| 2019 | 0.09 (0.06–0.15) | <0.001 |
| 2020 | 0.07 (0.04–0.11) | <0.001 |
| 2021 | 0.10 (0.07–0.15) | <0.001 |
| 2022 | 0.09 (0.06–0.11) | <0.001 |
